# Effect of specific mode electroacupuncture stimulation combined with NGF on dysphagia after ischemic stroke: study protocol for a pilot randomized controlled trial

**DOI:** 10.3389/fneur.2026.1697292

**Published:** 2026-03-18

**Authors:** Xiaoyang Yu, Ziqi Xuan, Zhaoxing Jia, Xuekang Niu, Yan Yan, Youmei Zhang, Lili Lin, Jingjing Li, Congcong Ma, Xianming Lin

**Affiliations:** 1 The Third Clinical Medical College, Zhejiang Chinese Medical University, Hangzhou, Zhejiang, China; 2 The Third Affiliated Hospital of Zhejiang Chinese Medical University, Hangzhou, Zhejiang, China; 3 Zhejiang Rehabilitation Medical Center, Hangzhou, Zhejiang, China

**Keywords:** blood–brain barrier, dysphagia after ischemic stroke, nerve growth factor, pilot randomized controlled trial, specific mode electroacupuncture stimulation, study protocol

## Abstract

**Background:**

Dysphagia is a major complication following ischemic stroke, but lacks effective treatments. Our previous studies show specific mode electroacupuncture stimulation (SMES) is an alternative approach to open the blood–brain barrier (BBB). Delivery of NGF across the BBB by SMES may be effective for treating dysphagia after ischemic stroke. The aim of this study is to assess the efficacy and safety of SMES combined with NGF for dysphagia after ischemic stroke.

**Methods:**

A total of 144 participants will be randomly assigned to an electroacupuncture (EA) + NGF group, an EA + placebo group, an acupuncture + NGF group, or an acupuncture + placebo group in a ratio of 1:1:1:1. Patients will receive 18 sessions of treatment for 6 weeks and a follow up of 6 weeks. The primary outcome is the Functional Oral Intake Scale (FOIS), and the secondary outcomes are the Dysphagia Outcome and Severity Scale (DOSS), the Penetration Aspiration Scale (PAS), the modified Rankin Scale (mRS), and the Modified Barthel Index (MBI). The functional near-infrared spectroscopy (fNIRS) will be applied to detect the changes in neurological function. Additionally, blinding and safety will also be assessed. All analysis will be performed based on intention-to-treat.

**Discussion:**

This trial will explore the feasibility and provide potential evidence for the effectiveness and safety of SMES combined with NGF for dysphagia after ischemic stroke.

**Clinical trial registration:**

https://www.clinicaltrials.gov/ct2/show/NCT06737549, identifier (NCT06737549).

## Introduction

Dysphagia is a major complication following acute stroke (1), with an incidence of 78% (2). Post-stroke dysphagia (PSD) can have a long-term impact on the dietary and nutritional intake of survivors, and even lead to pneumonia, or increase the hospitalization rate and mortality. Currently, non-pharmacological treatments, including dietary and nutritional interventions, behavioral treatment, dedicated oral health care, and peripheral or central neurostimulation strategies (3, 4), have demonstrated greater efficacy than pharmacological treatments for PSD. However, more evidence is needed to prove the effectiveness of non-pharmacological treatments for PSD (5). Pharmacological treatments for PSD, such as transient receptor potential cation channel subfamily V member 1 (TRPV-1) receptor agonists, angiotensin converting enzyme (ACE) inhibitors or dopaminergic agents, are generally administered only in research settings, due to the limited evidence available with regards to clinical endpoints (3, 4).

Nerve growth factor (NGF) is a neurotrophic substance that exhibits remarkable neurorepair and neuroprotective properties, displaying an active role in nourishing nerves, promoting neuronal survival, and inhibiting apoptosis (6). The advent of NGF offered a promising option for the treatment of central nervous system diseases. However, its actual clinical efficacy is less than satisfactory. The main issue lies in the fact that NGF, with a molecular weight of 13.4 kDa, is too large to cross the blood–brain barrier (BBB), preventing it from achieving effective blood concentrations in the central nervous system and limiting its application in central nervous system diseases (7). Therefore, one of the current focuses of neurological rehabilitation research is how to transport NGF and other large molecule neurotrophic drugs across the BBB to brain tissue, to achieve effective blood concentrations and exert their neurotrophic effects.

Previous research by our team has demonstrated that applying specific mode electroacupuncture stimulation (SMES) to the GV20 (Baihui) and GV26 (Shuigou) acupuncture points could open the BBB in rats (8). Through a series of studies, we have determined that the optimal parameters for SMES are 2/100 Hz, 3 mA, 6 s on and 6 s off (6–6 s), with a total stimulation duration of 40 min (8–10). Further research revealed that the effect of SMES on opening the BBB exhibits a time window, is dependent on the duration of stimulation, and is reversible (9, 11). Moreover, SMES-induced BBB opening does not cause adverse effects such as cerebral edema, glial cell activation, or neuronal apoptosis (9). The SMES applied at GV20 and GV26 acupoints has been shown to decrease the expression of tight junction proteins ZO-1 and occludin within the BBB, which can explain part of the potential mechanism of SMES opening the BBB (12). Additionally, SMES can induce NGF to cross the BBB, thereby improving motor and cognitive functions in rats during the recovery phase of middle cerebral artery occlusion/reperfusion (MCAO/R) (12). Another randomized controlled trial by our team, which is awaiting publication, investigating SMES combined with NGF for ischemic stroke, has shown significant therapeutic efficacy on motor and cognitive function and good safety (13).

These studies further consolidate our central hypothesis that the application of SMES to open the BBB will facilitate the delivery of exogenous NGF to achieve effective concentrations within the injured brain of post-stroke patients. We hypothesize that this approach can also benefit patients with PSD. Our team plans to conduct a pilot randomized controlled clinical trial on the treatment of dysphagia after ischemic stroke using SMES combined with NGF, to assess its effectiveness and safety.

To separately test the “BBB-opening carrier effect” of SMES, the “neuro-reparative effect” of NGF, their potential synergistic interaction, and to control for the non-specific effects of needling at the GV20 and GV26 acupoints themselves, we have adopted a 2 (SMES/Acupuncture) × 2 (NGF/Placebo) factorial, double-dummy design. This design aims to isolate and quantify the independent and combined contributions of each intervention component, providing a foundation for future mechanistic exploration and treatment optimization.

Functional near-infrared spectroscopy (fNIRS) can indirectly monitor cerebral cortical functional activity through changes in the concentrations of oxyhemoglobin (HbO2) and deoxyhemoglobin (HbR). Many fNIRS-based neuroimaging studies revealed that improved swallowing recovery in PSD is associated with upregulated activation patterns in the cortical swallowing network and strengthened functional integration among specific regions (14). We incorporated fNIRS into our study and integrated it with clinical efficacy assessments to investigate whether changes in swallowing function are associated with the therapeutic effects of SMES combined with NGF.

## Methods

### Study design

**Figure 1:**
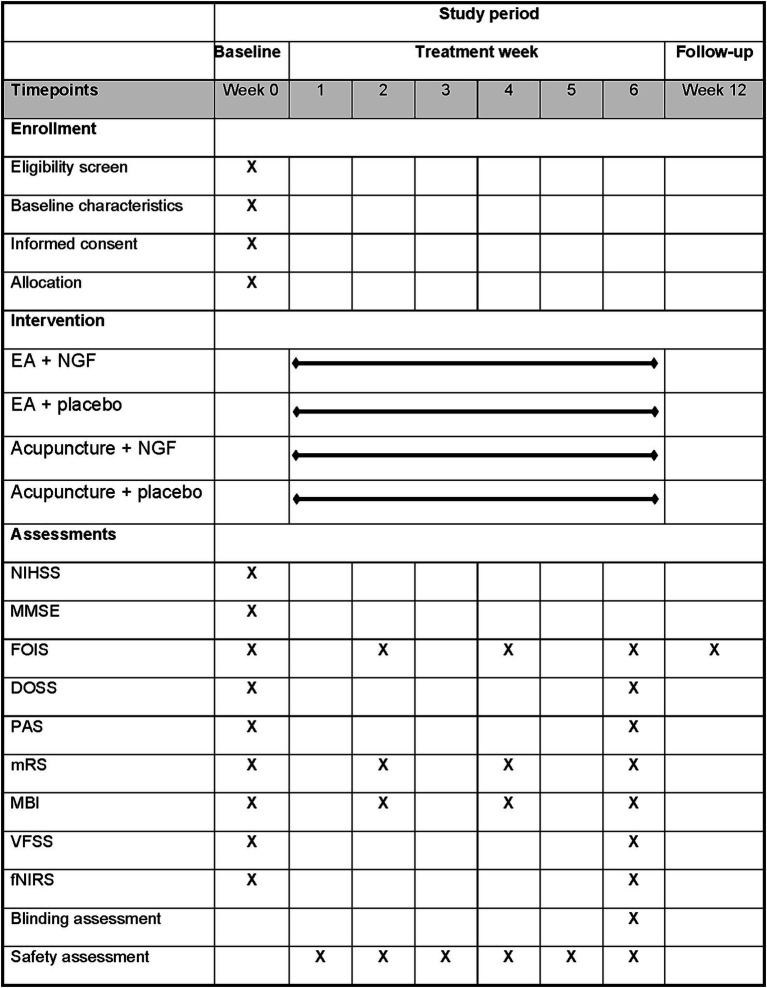
Schedule of enrollment, intervention, and assessment.

**Figure 2:**
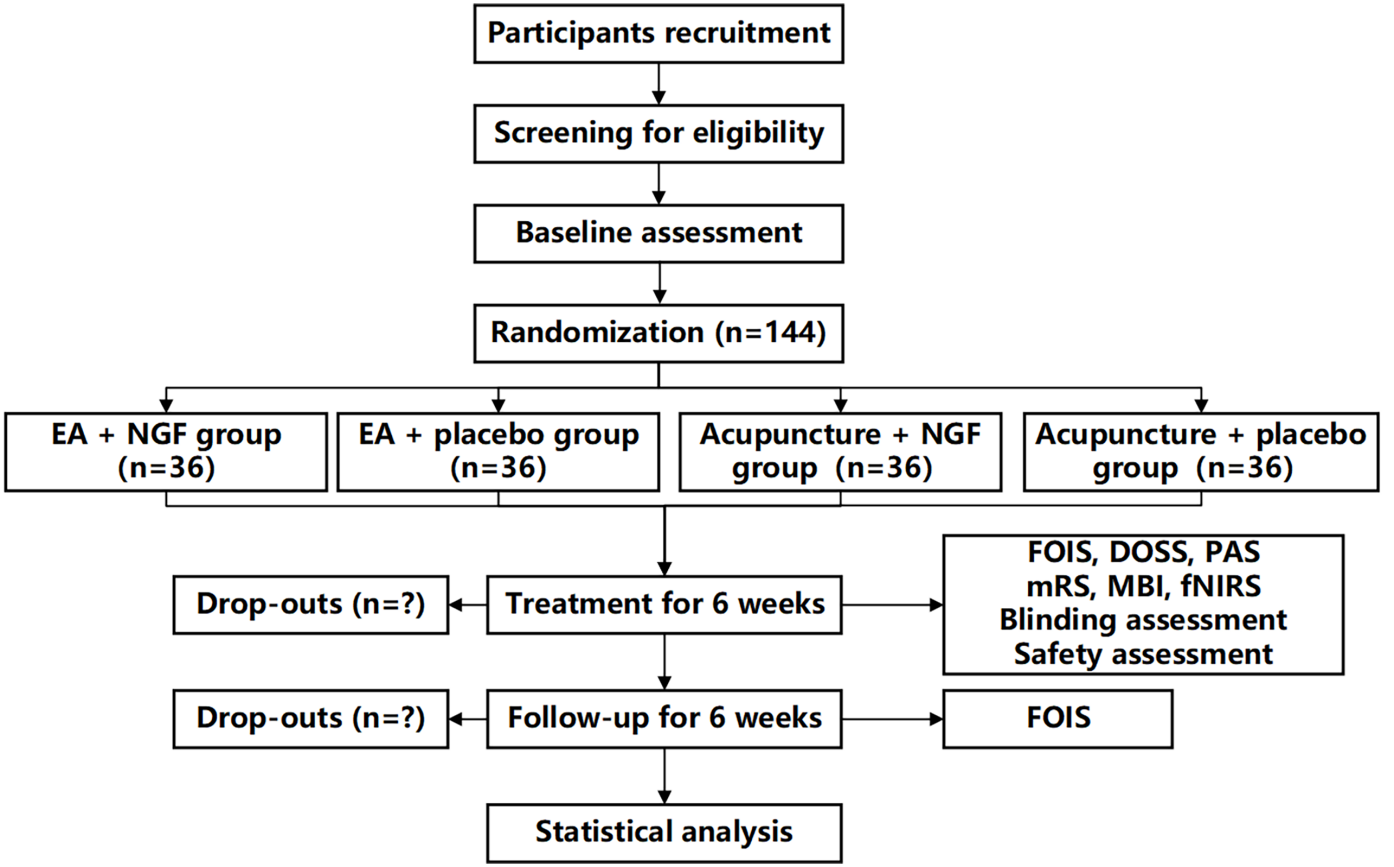
Flow chart of the study.

This study is a single-center, randomized, double-dummy, sham-controlled, four-arm parallel trial conducted at the Third Affiliated Hospital of Zhejiang Chinese Medical University. The schedule of enrolment, intervention, and assessment is shown in Figure 1. The flow chart is shown in Figure 2. The study protocol will be drafted in accordance with the guidelines of the Standard Protocol Items: Recommendations for Interventional Trials (SPIRIT) (15). The study conforms to the principles of the Declaration of Helsinki (16) and has been approved by the Medical Ethics Committee of the Third Affiliated Hospital of Zhejiang Chinese Medical University (ethical number: ZSLL-KY-2024-074-01). This trial has been registered at www.clinicaltrials.gov (NCT: NCT06737549) on December 12, 2024. Informed consent will be obtained from each participant before the performance of any study-specific procedure.

### Participants and recruitment

A total of 144 participants will be recruited through the notices posted in the hospital or on websites and social media (WeChat) from December 2024 to December 2027. The research assistants will be in charge of the recruitment, and neurological experts will be in charge of the diagnosis of the participants.

### Randomization and allocation concealment

One hundred forty-four eligible participants who have affirmed and signed informed consent will be randomly assigned in a ratio of 1:1:1:1 into the electroacupuncture (EA) + NGF group, the EA + placebo group, the acupuncture + NGF group or the acupuncture + placebo group, each with 36 participants, via a central randomization system, which is managed by a third-party mathematician outside the study and used to generate and conceal the allocation sequence. The randomization sequence will be generated in varying block sizes.

### Blinding

Participants, outcome assessors, and statisticians will remain blinded to treatment allocations. However, as the interventions (acupuncture or EA combined with either NGF or placebo) require direct physical contact with participants, the practitioners administering these interventions cannot be blinded. All participants will be treated separately to prevent communication. Except for acupuncturists, all relevant parties will be blinded to the intervention groups. Participants will be informed that they have an equal chance of allocation to each group before study participation.

### Participants

#### Inclusion criteria

Participants will be eligible if they:

(1) Have a first-ever ischemic stroke confirmed by Computed Tomography (CT) and/or Magnetic Resonance Imaging (MRI); (2) have an onset time ranging from 31 days to 90 days; (3) are aged 18 to 80 years, male or female; (4) have dysphagia confirmed by video fluoroscopic swallowing study (VFSS); (5) are able to accept and comply with acupuncture, EA treatment, gluteal intramuscular injection; (6) have already signed consent and exhibit willingness to participate in the trial.

#### Exclusion criteria

Participants will be excluded if they:

(1) Had dysphagia before this ischemic stroke onset or dysphagia not caused by ischemic stroke; (2) have severe cognitive impairment; (3) have a history of multiple strokes, craniocerebral surgery, or cerebral space-occupying lesions; (4) have severe cardiac, hepatic, or renal dysfunction, or other abnormal test results that make the patient unsuitable for participation in this study; (5) have severe neurological deficits prior to ischemic stroke, such as visual and auditory impairments, aphasia, agnosia, severe hemiplegia, and other conditions; (6) are women who are lactating, pregnant or planning pregnancy; (7) have a history of needle fainting or with skin infections at the acupuncture sites; (8) are carriers of a cardiac pacemaker; (9) have a known allergy to NGF; (10) are unable to undergo VFSS; (11) have taken medications that may alter cortical excitability within the past 2 months; (12) are currently participating in other clinical trials or have participated in clinical trials that ended within the last 3 months.

### Intervention

To ensure consistency in interventions, qualified acupuncturists with certifications will be selected and trained. Participants in 4 groups will receive EA/acupuncture and NGF/placebo intervention for 6 weeks, three sessions per week, in inpatient wards and outpatient departments. The locations of all acupoints will be based on the World Health Organization (WHO) Standard Acupuncture Locations (17). The following materials will be used: disposable sterile acupuncture needles of two different specifications (0.25 mm × 40 mm, 0.25 mm × 25 mm; Hwato brand; Suzhou Medical Supplies Factory Co. LTD, Suzhou, China); an acupuncture point nerve stimulator (HANS-200A, Nanjing Jisheng, Ltd., China), a specially made relay (cycled power to the electrode for 6 s on and 6 s off), 20 μg mouse nerve growth factor (mNGF) (NO. S20060051, Jinlujie, Hiteck Biopharmaceutical Co., Ltd., Wuhan, China); 5 mL sterile water for injection (NO. H33022534, Ruixin Pharma, Lishui, China); and 10 mL 0.9% sodium chloride injection (NO. H20043271, China Otsuka Pharmaceutical Co., Ltd., China).

Additionally, swallowing training for dysphagia and conventional treatment for ischemic stroke will be allowed, which are included in the Chinese Guidelines for the Clinical Management of Cerebrovascular Diseases (Second Edition) (18).

### EA + NGF group

**Figure 3:**
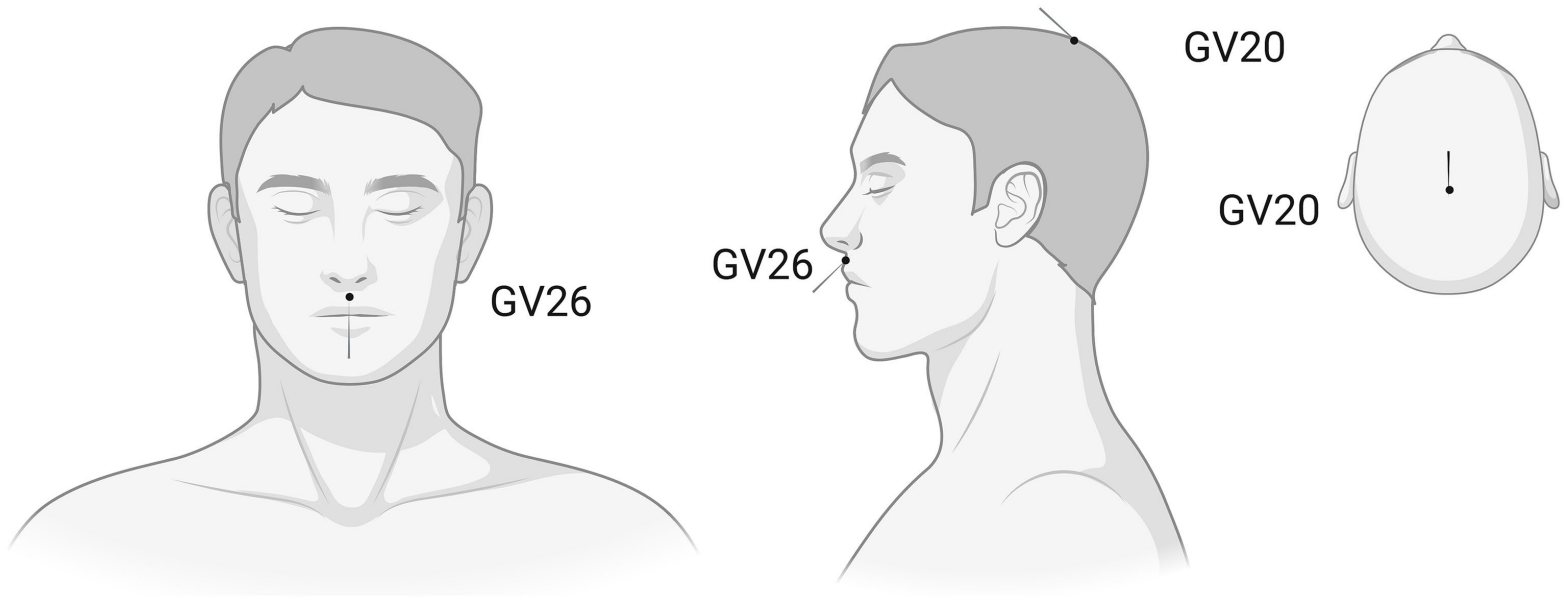
Location and needling direction diagram of GV20 (Baihui) and GV26 (Shuigou). This image was generated using BioRender software (http://www.biorender.com).

A 20 μg dose of mNGF will be dissolved in 2 mL of sterile water for intramuscular injection into the gluteus maximus. Thirty minutes after injection, participants will receive EA treatment. The acupoints GV20 and GV26 will be acupunctured (Figure 3). Participants will lie in the supine position and relax. Prior to acupuncture, 75% alcohol pads will be used to sterilize the skin around the acupuncture points. For GV20, an acupuncture needle (0.25 mm × 40 mm) will be inserted transversely toward the central point of the posterior hairline to a depth of 15–20 mm. For GV26, an acupuncture needle (0.25 mm × 25 mm) will be inserted toward the nasal septum to a depth of 5–10 mm. Acupoints will be stimulated manually until participants feel soreness, distension or heaviness (the reaction of “De Qi”). Then, the needles will be stimulated using an acupuncture point nerve stimulator at a frequency of 2/100 Hz and an intensity tolerable to the participant (targeting 3.0 mA) for 40 min. A specially made relay cycles power to the electrodes in a 6-s on/6-s off pattern. The needles at both acupoints will be retained for 40 min. Participants will receive 18 treatment sessions, 3 times per week for 6 weeks.

### EA + placebo group

Participants will receive an intramuscular injection of 2 mL of 0.9% sodium chloride into the gluteus maximus. Thirty minutes after injection, participants will receive EA treatment following the same protocol as the EA + NGF group. Participants will receive 18 treatment sessions, 3 times per week for 6 weeks.

### Acupuncture + NGF group

Participants will receive an intramuscular injection of 20 μg mNGF into the gluteus maximus, as in the EA + NGF group. Thirty minutes after injection, participants will receive acupuncture treatment. The treatment protocol will be similar to that of the EA + NGF group, except that the needles will be connected to an acupuncture point nerve stimulator without actual current output. The device will display a frequency of 2/100 Hz and a current strength of 3.0 mA for 40 min, and a specially made relay will cycle in a 6-s on / 6-s off pattern. When switched on, the apparatus produces the same indicator lights and sounds as the active device but delivers no electrical current. The needles at both points will be retained for 40 min. Participants will receive 18 treatment sessions, 3 times per week for 6 weeks.

### Acupuncture + placebo group

Patients will receive an intramuscular injection of 2 mL of 0.9% sodium chloride into the gluteus maximus. Thirty minutes after injection, they will receive acupuncture treatment following the same protocol as the acupuncture + NGF group. The needles at both points will be retained for 40 min. Participants will receive 18 treatment sessions, 3 times per week for 6 weeks.

### Swallowing training

Prior to initiating swallowing training, a qualified rehabilitation therapist conducts a swallowing assessment of the participant. Based on the evaluation results, rehabilitation goals are established, and an individualized swallowing training plan is formulated. The training program is implemented by a qualified rehabilitation therapist according to the severity of dysphagia. Swallowing training includes direct training techniques (compensatory methods, behavioral maneuvers), and indirect training techniques (orofacial sensory-motor training, airway protection maneuvers) (19). The swallowing training will be administered by a qualified rehabilitation therapist for about 30 min per day, 5 days a week, over 6 weeks.

### Outcomes

#### Primary outcome

The primary outcome is the Functional Oral Intake Scale (FOIS) (20), which reflects the overall intake capability based on swallowing function. The FOIS is a 7-point ordinal scale with a score range of 1–7. Oral intake ability is measured in terms of intake method and food type. Higher scores indicate better oral intake ability. Assessment time frames: at baseline; at week 2, 4, 6, and 12. Week 6 post-intervention is the primary endpoint.

#### Secondary outcomes

Secondary outcomes will include the following:

The Dysphagia Outcome and Severity Scale (DOSS): The DOSS integrates swallowing efficiency and safety. The DOSS scores range from 1 to 7 with 7 grades, and lower scores indicate more severe dysphagia (21). Assessment time frames: at baseline; at week 6.

The Penetration Aspiration Scale (PAS): The PAS focuses on the safety of swallowing. The PAS scores range from 1 to 8 with 8 grades, and higher scores indicate higher risk of penetration and aspiration (22). Assessment time frames: at baseline; at week 6.

The Modified Rankin Scale (mRS): The mRS is a valid and clinically relevant instrument for assessing recovery from stroke (23), and the mRS scores range from 0 to 5 with 6 grades. Assessment time frames: at baseline; at week 2, 4, 6.

The Modified Barthel Index (MBI): The MBI (24) is a common scale for evaluating activities of daily living (ADL), with a total score of 100 points. Assessment time frames: at baseline; at week 2, 4, 6.

**Figure 4:**
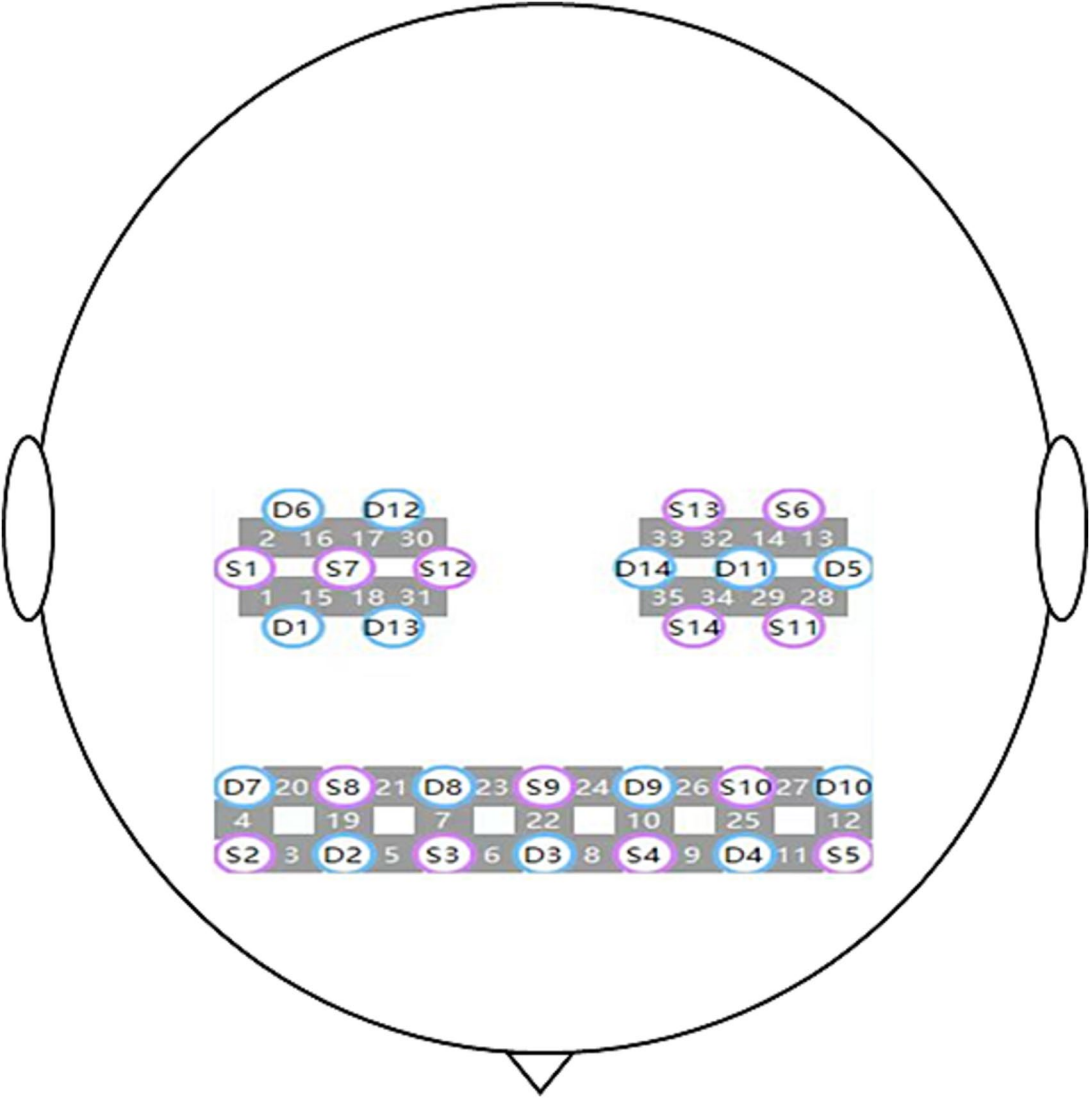
Regions of interest and the channel setting.

Oxygenated hemoglobin and deoxygenated hemoglobin in cerebral cortex: An fNIRS device with wavelengths of 730 and 850 nm (Danyang Hui Chuang Medical Equipment Co., Ltd.) will be used to detect changes in HbO2 and HbR in swallowing-related brain areas during rest and voluntary swallowing. A total of 35 channels will be distributed across the right and left prefrontal cortices (RPFC/LPFC), motor areas (RM1/LM1), somatosensory areas (RS1/LS1), and pre-motor and supplementary motor areas (RPM/LPM). The sampling rate of the fNIRS system will be set to 11 Hz. According to the anatomical positions defined by the international 10–20 system, the probes will be placed on the scalp surface of the subjects based on four reference points (nasal root, external occipital protuberance, left pre-auricular point, right pre-auricular point). A schematic diagram of the probe and channel arrangement is shown in Figure 4 (14). Participants will be seated in a quiet fNIRS assessment room to reduce the impact of noise.

During the formal test, the fNIRS device gives start and stop cues at corresponding time points, and the subjects are required to perform swallowing tasks according to the instructions issued by the device. The entire test process consists of 3 identical task blocks, each of which includes 30 s of voluntary swallowing task and 30 s of rest time. During the voluntary swallowing task, the subjects maintain a natural and relaxed state, close their eyes, and perform the repetitive saliva swallowing test task at their own pace.

**Figure 5:**
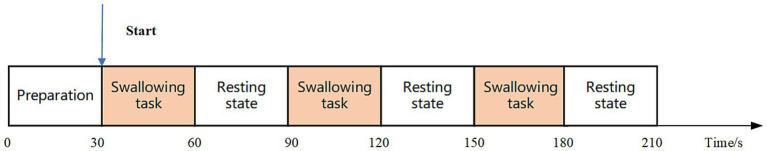
The experimental procedure.

During the rest period, the subjects are in a natural and relaxed state, close their eyes, remain quiet, and avoid thinking about things unrelated to the experimental task. The schematic diagram of the fNIRS voluntary swallowing task paradigm is shown in Figure 5 (25). The room is kept noise-free and free from external interference. Throughout the entire test process, the subjects always maintain a natural and relaxed state, close their eyes, and complete the corresponding tasks according to the instructions issued by the device. The head and body positions should be kept as still as possible to avoid affecting the fNIRS signal acquisition. Assessment time frames: at baseline; at week 6.

### Blinding assessment

The Bang’s Blinding Index (26) will be used to assess the adequacy of blinding at week 6.

### Safety assessment

Adverse reactions or adverse events will be recorded in detail, including the time of occurrence of adverse events, symptoms and signs, laboratory examination results, treatment to relieve adverse events, follow-up of adverse events, duration and severity of adverse events. For any serious adverse event occurring during the study (including events causing disability or impact on work ability, life-threatening events or death, etc.), the investigator must take appropriate treatment measures immediately and report to the ethics committee within 24 h or no later than the second working day.

### Data management and monitoring

Data collection and randomization will be handled by the research assistants. The double-input method will be used for data entry, and all data related to patients will be stored confidentially. All researchers and acupuncturists will take a training course before performing the study. The whole process of this trial will be conducted under the supervision of three levels of monitors. The first level of monitors, composed of researchers certified in acupuncture, will be responsible for overseeing the entire study. The second level of monitors, composed of researchers from the ethics committee, will be responsible for study supervision. The third level of monitors, composed of data supervisors, will ensure data authenticity. We have incorporated a comprehensive safety monitoring plan into the protocol: (1) Data Safety Monitoring Board (DSMB): An independent DSMB comprising a neurologist, a stroke rehabilitation specialist, and a biostatistician will be established prior to trial commencement. (2) Pre-specified Stopping Rules: Clear safety stopping rules have been defined, including (a) a significant increase in the rate of symptomatic intracranial hemorrhage beyond expected rates, (b) occurrence of successive serious adverse events related to neurological worsening (increase in NIHSS score by ≥4 points), or (c) a judgment by the DSMB that overall risks outweigh benefits.

### Statistical methods

#### Sample size

This pilot study aims to assess the effectiveness and safety of SMES combined with NGF for dysphagia after ischemic stroke and determine the feasibility of a further definitive trial. To ensure that the clinical trial data have sufficient statistical power, the minimum sample size of each group of subjects should not be less than 30 participants (27). To account for an anticipated 20% dropout rate, the sample size was inflated to 36 participants per group, resulting in a total enrollment of 144 participants.

### Statistical analysis

The overall dropout rate and the dropout rate due to adverse events will be calculated for each group. It will be statistically determined whether each group received the treatment as specified in the study protocol in terms of duration and number of sessions, and the number and percentage of subjects with compliance < 80% will be calculated. Efficacy analysis will be performed in the full analysis set. Participants who have been randomized and have received at least one treatment session will be included in the full analysis set (FAS). All hypothesis tests will be two-sided, and *p*-value < 0.05 will be considered to indicate statistical significance of the tested difference. For ordinal outcomes (FOIS, DOSS, PAS, and mRS), non-parametric tests (Kruskal–Wallis *H* test for group comparisons, with post-hoc Mann–Whitney *U* tests and alpha adjustment) will be used for cross-sectional analyses. For longitudinal ordinal outcomes, generalized estimating equations (GEE) with an appropriate link function (e.g., logit) and working correlation matrix will be the primary method for analyzing these outcomes across multiple time points (FOIS, mRS). The normality of continuous data (MBI, fNIRS metrics) will be assessed using the Shapiro–Wilk test. Provided that parametric assumptions are met, (repeated-measures) ANOVA will be applied. Should the assumptions be violated, non-parametric alternatives (e.g., Kruskal–Wallis test, Friedman test) will be used. The study results will include three-factor (EA factor/NGF factor/time factor) repeated-measure data, with measurement data analyzed using three-factor repeated-measures analysis of variance and ordinal data analyzed using the generalized estimating equation (GEE) to test for differences. The Bonferroni method will be used to adjust for the significance of multiple comparisons.

For the analysis of fNIRS data, preprocessing will be conducted using the NirSpark software package (HuiChuang, China). Initial inspection of raw signals will be performed by an expert to exclude low-quality segments. Motion artifacts in each channel will be corrected through spline interpolation, targeting locally confined signal disruptions. Additionally, a band-pass filter (0.01–0.2 Hz) will be applied to attenuate physiological noise. Subsequent conversion to hemoglobin concentration changes in HbO2 and HbR concentrations will be carried out via the modified Beer–Lambert law (28). The hemodynamic response function (HRF) will be defined with a baseline period of 0–30 s and a task period spanning 30–90 s, extending the analysis window to 210 s. Generalized linear modeling will be applied to the HbO₂ and HbR time-series from each preprocessed dataset. Finally, hemoglobin time series covering 210 s will be extracted for every participant.

The hemoglobin time series for each channel pair will be analyzed using Pearson correlation. Given the normal distribution properties of the z-matrix, the resulting correlation coefficients (*r*) will be converted to *z*-scores using Fisher’s r-to-z transformation prior to further analysis. Based on Brodmann area (BA) classifications, measurement channels will be grouped into four regions: PFC, PM, S1, and M1. Taking into account the bilateral structure of the cerebral hemispheres, a total of eight distinct regions will be defined. An 8 × 8 functional connectivity matrix will then be constructed by independently averaging the z-scores, which will allow for the comparison of functional connectivity across networks.

### Ethics and dissemination

This study has been approved by the Medical Ethics Committee of the Third Affiliated Hospital of Zhejiang Chinese Medical University (ethical number: ZSLL-KY-2024-074-01). Informed consent will be obtained from each patient before the performance of any study-specific procedure. Personal information about potential and enrolled participants will be protected and strictly confidential before, during, and after the trial. Data of the results without personal information from this study will be planned to disseminate in conferences or peer-reviewed publications.

## Discussion

This manuscript presents the design of a randomized controlled pilot trial testing the effectiveness and safety of SMES combined with NGF to improve dysphagia after ischemic stroke. To the best of our knowledge, there is no published literature on the effectiveness of SMES combined with NGF for dysphagia after ischemic stroke.

Dysphagia, one of the most common complications of stroke, is characterized by impaired swallowing efficacy (leading to malnutrition and dehydration) and compromised swallowing safety (increasing risks of aspiration and aspiration pneumonia) (1). Even with the most effective rehabilitation strategies, satisfactory outcomes may not always be achievable. In this design, we apply an alternative approach for NGF delivery to enhance the clinical efficiency. Based on our previous achievements, SMES is a convenient and effective approach to open the BBB in a controllable and reversible manner. Building on standard swallowing training, SMES may facilitate NGF delivery across the BBB and promote swallowing network plasticity. The concept of opening the BBB is absent in traditional acupuncture theory; thus, SMES represents an innovation beyond conventional acupuncture. SMES holds significant promise for drug delivery across the BBB to treat brain diseases (12, 29).

There are numerous clinical studies on NGF therapy for peripheral nerve injury (30). However, its application in central nervous system disorders has been limited by the BBB. By opening the BBB, SMES may enable NGF to reach brain targets, potentially improving swallowing function after stroke. Nevertheless, randomized controlled trials (RCTs) are needed to validate this combined intervention. The present study aims to provide preliminary evidence for its clinical application.

Regarding outcome measures, the primary outcome is the FOIS, a validated tool for assessing global swallowing capacity. Secondary outcomes include the DOSS, which evaluates swallowing efficiency and safety; the PAS, which focuses on aspiration risk (19); the mRS, which measures post-stroke functional recovery; and the MBI, which assesses activities of daily living.

Multimodal findings from clinical, electrophysiological, and imaging studies suggest that dysphagia recovery after stroke involves neuroplastic changes in the contralesional hemisphere across sensory, motor, and white matter domains (31–39). This body of evidence supports a compensatory model of contralesional neuroplasticity as the driving mechanism for recovery (1). fNIRS and electroencephalography (EEG) are two convenient brain function imaging techniques that have attracted significant attention in recent years and have yielded numerous significant findings on the mechanism of acupuncture’s effects on the brain (14, 40–42). Currently, the application of EEG in the study of PSD remains limited, but many fNIRS studies have proved that the effect of acupuncture can upregulate activation patterns in the cortical swallowing network and strengthen functional integration among specific regions (14). This study is not focused on the direct cerebral effects of acupuncture. Instead, fNIRS was chosen as the research method to examine the swallowing network recovery induced by NGF. In terms of study design, the four-arm study can highlight the interaction effect between SMES and NGF, and distinguish it from the independent effect of acupuncture at the GV20 and GV26 acupoints.

In China, a significant proportion of PSD patients have prior acupuncture experience, making patient blinding challenging (43). Thus, conventional acupuncture rather than sham acupuncture will serve as the control to optimize blinding feasibility.

There are some limitations to our study. First, as a pilot trial, the sample size is relatively small, which may increase susceptibility to bias. Second, acupuncturists could not be blinded due to the nature of the intervention, potentially introducing performance bias. Third, future studies should incorporate pharmacokinetic sub-studies to optimize the timing between NGF administration and SMES stimulation. Fourth, the SMES parameters (2/100 Hz, 3 mA, 6 s-on/6 s-off) were derived from rodent models. Given biophysical differences between species (e.g., impedance, skull geometry), the intracranial current density in humans requires empirical validation. Future research should use computational modeling, neuroimaging (e.g., dynamic contrast-enhanced MRI), or electrophysiological monitoring for systematic dose-finding. Fifth, while the factorial design distinguishes SMES-specific effects from traditional acupuncture, any observed superiority of SMES + NGF would require confirmation in future trials with strict sham controls and biomarkers before attributing it definitively to BBB-mediated NGF delivery. Sixth, a negative or null result could reflect two distinct possibilities: (1) lack of biological efficacy of the combined intervention, or (2) suboptimal temporal alignment between NGF availability and BBB opening, despite a potentially efficacious mechanism. The current design cannot distinguish between these. Thus, a negative result would highlight the need for further pharmacokinetic-pharmacodynamic studies to optimize intervention timing before concluding on biological plausibility.

## References

[1] Labeit B Michou E Hamdy S Trapl-Grundschober M Suntrup-Krueger S Muhle P . The assessment of dysphagia after stroke: state of the art and future directions. Lancet. Neurol. (2023) 22:858–70. doi: 10.1016/S1474-4422(23)00153-9, 37596008

[2] Rofes L Muriana D Palomeras E Vilardell N Palomera E Alvarez-Berdugo D . Prevalence, risk factors and complications of oropharyngeal dysphagia in stroke patients: a cohort study. Neurogastroenterol Motil. (2018) 30:e13338. doi: 10.1111/nmo.13338, 29573064

[3] Dziewas R Michou E Trapl-Grundschober M Lal A Arsava EM Bath PM . European stroke organisation and european society for swallowing disorders guideline for the diagnosis and treatment of post-stroke dysphagia. Eur Stroke J. (2021) 6:LXXXIX–CXV. doi: 10.1177/23969873211039721 34746431 PMC8564153

[4] Labeit B Michou E Trapl-Grundschober M Suntrup-Krueger S Muhle P Bath PM . Dysphagia after stroke: research advances in treatment interventions. Lancet Neurol. (2024) 23:418–28. doi: 10.1016/S1474-4422(24)00053-X 38508837

[5] Bath PM Lee HS Everton LF. Swallowing therapy for dysphagia in acute and subacute stroke. Cochrane Database Syst Rev. (2018) 10:CD000323. doi: 10.1002/14651858.CD000323.pub3, 30376602 PMC6516809

[6] Gutiérrez-Fernández M Fuentes B Rodríguez-Frutos B Ramos-Cejudo J Vallejo-Cremades MT Díez-Tejedor E. Trophic factors and cell therapy to stimulate brain repair after ischaemic stroke. J Cell Mol Med. (2012) 16:2280–90. doi: 10.1111/j.1582-4934.2012.01575.x 22452968 PMC3823421

[7] Weissmiller AM Wu C. Current advances in using neurotrophic factors to treat neurodegenerative disorders. Transl Neurodegener. (2012) 1:14. doi: 10.1186/2047-9158-1-14, 23210531 PMC3542569

[8] Zhang J Lin X Zhou H Chen Y Xiao S Jiao J . Electroacupuncture: a new approach to open the blood-brain barrier in rats recovering from middle cerebral artery occlusion. Acupunct Med. (2018) 36:377–85. doi: 10.1136/acupmed-2017-011496, 29903719 PMC6287560

[9] Zhang S Gong P Zhang J Mao X Zhao Y Wang H . Specific frequency electroacupuncture stimulation transiently enhances the permeability of the blood-brain barrier and induces tight junction changes. Front Neurosci. (2020) 14:582324. doi: 10.3389/fnins.2020.582324 33122995 PMC7573286

[10] Ma C Gan L Wang H Ren L Lin Y Zhao Y . Transcriptomic analysis of rat cerebral cortex reveals the potential mechanism of electroacupuncture opening blood brain barrier. Front Neurosci. (2022) 16:834683. doi: 10.3389/fnins.2022.834683 35281512 PMC8908321

[11] Lin Y Gan L Ren L Ma C Dai M Qian K . Acupuncture with specific mode electro-stimulation effectively and transiently opens the BBB through shh signaling pathway. Neuroreport. (2023) 34:873–86. doi: 10.1097/WNR.0000000000001970 37942738

[12] Dai M Qian K Ye Q Yang J Gan L Jia Z . Specific mode electroacupuncture stimulation mediates the delivery of NGF across the hippocampus blood-brain barrier through p65-VEGFA-TJs to improve the cognitive function of MCAO/r convalescent rats. Mol Neurobiol. (2025) 62:1451–66. doi: 10.1007/s12035-024-04337-8, 38995444 PMC11772513

[13] Dai M Zhao Y Jia Z Xu S Xu N Wu X . Effect of specific mode electroacupuncture stimulation combined with NGF during the ischaemic stroke: study protocol for a randomized controlled trial. Clinics. (2024) 79:100451. doi: 10.1016/j.clinsp.2024.100451, 39033586 PMC11325668

[14] Fu X Li H Yang W Li X Lu L Guo H . Electroacupuncture at HT5 + GB20 promotes brain remodeling and significantly improves swallowing function in patients with stroke. Front Neurosci. (2023) 17:1274419. doi: 10.3389/fnins.2023.1274419 38027487 PMC10656700

[15] Chan A Tetzlaff JM Altman DG Laupacis A Gøtzsche PC Krleža-Jerić K . SPIRIT 2013 statement: defining standard protocol items for clinical trials. Ann Intern Med. (2013) 158:200–7. doi: 10.7326/0003-4819-158-3-201302050-00583 23295957 PMC5114123

[16] World Medical Association. World medical association declaration of Helsinki: ethical principles for medical research involving human subjects. JAMA. (2013) 310:2191–4. doi: 10.1001/jama.2013.281053 24141714

[17] Organization WH. WHO Standard Acupuncture Point Locations in the Western Pacific Region. (2008). p. 255.

[18] Chinese Stroke Association. Chinese Guidelines for the Clinical Management of Cerebrovascular Diseases. 2nd ed. Beijing, China: People’s Medical Publishing House (2023).

[19] Dai M Qiao J Shi Z Wei X Chen H Shen L . Effect of cerebellar transcranial magnetic stimulation with double-cone coil on dysphagia after subacute infratentorial stroke: a randomized, single-blinded, controlled trial. Brain Stimul. (2023) 16:1012–20. doi: 10.1016/j.brs.2023.05.023, 37301470

[20] Crary MA Mann GDC Groher ME. Initial psychometric assessment of a functional oral intake scale for dysphagia in stroke patients. Arch Phys Med Rehabil. (2005) 86:1516–20. doi: 10.1016/j.apmr.2004.11.049, 16084801

[21] O'Neil KH Purdy M Falk J Gallo L. The dysphagia outcome and severity scale. Dysphagia. (1999) 14:139–45. doi: 10.1007/PL00009595, 10341109

[22] Rosenbek JC Robbins JA Roecker EB Coyle JL Wood JL. A penetration-aspiration scale. Dysphagia. (1996) 11:93–8. doi: 10.1007/BF00417897, 8721066

[23] Banks JL Marotta CA. Outcomes validity and reliability of the modified Rankin scale: implications for stroke clinical trials: a literature review and synthesis. Stroke. (2007) 38:1091–6. doi: 10.1161/01.STR.0000258355.23810.c6, 17272767

[24] Shah S Vanclay F Cooper B. Improving the sensitivity of the barthel index for stroke rehabilitation. J Clin Epidemiol. (1989) 42:703–9. doi: 10.1016/0895-4356(89)90065-6, 2760661

[25] Wen X Peng J Zhu Y Bao X Wan Z Hu R . Hemodynamic signal changes and functional connectivity in acute stroke patients with dysphagia during volitional swallowing: a pilot study. Med Phys. (2023) 50:5166–75. doi: 10.1002/mp.16535 37314082

[26] Bang H Ni L Davis CE. Assessment of blinding in clinical trials. Control Clin Trials. (2004) 25:143–56. doi: 10.1016/j.cct.2003.10.016, 15020033

[27] Browne RH. On the use of a pilot sample for sample size determination. Stat Med. (1995) 14:1933–40. doi: 10.1002/sim.4780141709, 8532986

[28] Li H Fu X Lu L Guo H Yang W Guo K . Upper limb intelligent feedback robot training significantly activates the cerebral cortex and promotes the functional connectivity of the cerebral cortex in patients with stroke: a functional near-infrared spectroscopy study. Front Neurol. (2023) 14:1042254. doi: 10.3389/fneur.2023.1042254 36814999 PMC9939650

[29] Ma C Ye Q Qian K Dai M Gan L Yang J . Anti-glioma effect of paclitaxel mediated by specific mode electroacupuncture stimulation and the related role of the hedgehog pathway. Brain Res Bull. (2024) 213:110985. doi: 10.1016/j.brainresbull.2024.110985 38806118

[30] Yan-rong L Qiang L Yuan T. Meta-analysis of mNGF therapy for peripheral nerve injury: a systematic review. Chin J Traumatol. (2012) 15:86–91. doi: 10.3760/cma.j.issn.1008-1275.2012.02.004 22480671

[31] Domin M Mihai GP Platz T Lotze M. Swallowing function in the chronic stage following stroke is associated with white matter integrity of the callosal tract between the interhemispheric s1 swallowing representation areas. NeuroImage Clinical. (2022) 35:103093. doi: 10.1016/j.nicl.2022.103093 35772193 PMC9253494

[32] Hamdy S Aziz Q Rothwell JC Crone R Hughes D Tallis RC . Explaining oropharyngeal dysphagia after unilateral hemispheric stroke. Lancet. (1997) 350:686–92. doi: 10.1016/s0140-6736(97)02068-0 9291902

[33] Hamdy S Aziz Q Rothwell JC Power M Singh KD Nicholson DA . Recovery of swallowing after dysphagic stroke relates to functional reorganization in the intact motor cortex. Gastroenterology. (1998) 115:1104–12. doi: 10.1016/s0016-5085(98)70081-2 9797365

[34] Suntrup-Krueger S Ringmaier C Muhle P Wollbrink A Kemmling A Hanning U . Randomized trial of transcranial direct current stimulation for poststroke dysphagia. Ann Neurol. (2018) 83:328–40. doi: 10.1002/ana.25151, 29350775

[35] Ko EJ Choi KH Kwon SU. The relationship between leukoaraiosis involving contralateral corticobulbar tract and dysphagia in patients with acute unilateral corona radiata infarction with corticobulbar tract involvement. Dysphagia. (2019) 34:654–64. doi: 10.1007/s00455-018-9963-y 30465078

[36] Lee WH Lim MH Seo HG Seong MY Oh B Kim S. Development of a novel prognostic model to predict 6-month swallowing recovery after ischemic stroke. Stroke. (2020) 51:440–8. doi: 10.1161/STROKEAHA.119.027439 31884906

[37] Im S Han YJ Kim S Yoon M Oh J Kim Y. Role of bilateral corticobulbar tracts in dysphagia after middle cerebral artery stroke. Eur J Neurol. (2020) 27:2158–67. doi: 10.1111/ene.14387 32524719

[38] Jang SH Kwak SY Chang CH Jung YJ Kim J Kim SH . Prognostic prediction of dysphagia by analyzing the corticobulbar tract in the early stage of intracerebral hemorrhage. Dysphagia. (2020) 35:985–92. doi: 10.1007/s00455-020-10093-3 32040613

[39] Cabib C Nascimento W Rofes L Arreola V Tomsen N Mundet L . Neurophysiological and biomechanical evaluation of the mechanisms which impair safety of swallow in chronic post-stroke patients. Transl Stroke Res. (2020) 11:16–28. doi: 10.1007/s12975-019-00701-2 30941716

[40] Yu H Li F Liu J Liu D Guo H Wang J . Evaluation of acupuncture efficacy in modulating brain activity with periodic-aperiodic EEG measurements. IEEE Trans Neural Syst Rehabil Eng. (2024) 32:2450–9. doi: 10.1109/TNSRE.2024.3421648, 38949930

[41] Rao W Xu M Wang H Hua W Guo J Zhang Y . Acupuncture state detection at zusanli (ST-36) based on scalp EEG and transformer. IEEE J Biomed Health Inform. (2025) 29:4023–34. doi: 10.1109/JBHI.2025.3540924, 40031811

[42] Yu H Zeng F Liu D Wang J Liu J. Neural manifold decoder for acupuncture stimulations with representation learning: an acupuncture-brain interface. IEEE J Biomed Health Inform. (2025) 29:4147–60. doi: 10.1109/JBHI.2025.3530922 40031188

[43] Guo H Pan X Zheng Y Yang X Xu H Zhang Y . Current state of research on acupuncture for the treatment of post-stroke dysphagia: a scoping review. Front Neurosci. (2024) 18:1391576. doi: 10.3389/fnins.2024.1391576 39211435 PMC11357938

